# Minimal surgery achieved good visual acuity in selected patients with magnetic intravitreal foreign body and traumatic cataract

**DOI:** 10.1186/s12886-019-1065-6

**Published:** 2019-02-19

**Authors:** Zhitao Su, Panpan Ye, Jijian Lin, Li Zhang, Xiaodan Huang

**Affiliations:** Eye Center, Second Affiliated Hospital, School of Medicine, Zhejiang University, No. 88 Jiefang Rd, Hangzhou, 310009 China

**Keywords:** Intravitreal foreign body, Traumatic cataract surgery, IOL implantation, Minimal surgery

## Abstract

**Background:**

To explore minimal surgery in selected patients with intravitreal foreign body (IVFD) and traumatic cataract.

**Methods:**

Twelve eyes of 12 patients with small ferrous IVFD and traumatic cataract without endophthalmitis, retinal injury and secondary glaucoma, between September 2015 and March 2017 were retrospectively analyzed. Primary removal of IVFD was performed by external magnetic extraction through the pars plana incision. Secondary removal of traumatic cataract by phacoemulsification and intraocular lens (IOL) implantation with or without anterior vitrectomy were performed. Patients were followed up at 1 day, 1 week, 1 month, 3 months, 6 months and 12 months after surgery.

**Results:**

All patients were male with a mean age of 32 years old. All IVFDs were successfully removed without retinal injury. Two to 6 months later, the traumatic cataract was successfully removed by phacoemulsification combined with IOL implantation in the capsule bag in 10 patients. Anterior vitrectomy was implied in 2 patients with large posterior capsule rupture, and the IOLs were placed in the ciliary sulcus. Best-corrected visual acuity ranged from hand movement to 20/100 before surgery and improved ranging from 20/32 to 20/20 at the final follow-up. The IOLs were well centered. Complications such as secondary glaucoma, endophthalmitis and retinal detachment were not found.

**Conclusions:**

Primary removal of small ferrous IVFD by external magnetic extraction followed by secondary cataract removal and IOL implantation is an appropriate choice. Minimal surgery may obtain good visual outcome without complications in selected patients.

## Background

Among patients with posterior segment intraocular foreign body (IOFB) and traumatic cataract, visual rehabilitation poses a unique challenge to ophthalmic surgeons. With the development of surgical techniques and instruments, there is an increasing trend toward performing pars plana vitrectomy (PPV) and simultaneous cataract extraction in the management of these patients [1–3]. However, various potential complications of PPV have been reported [4–6]. Ferrous intravitreal foreign body (IVFB), which did not damage the retina can be successfully removed by external magnetic extraction without PPV [7]. In the absence of increased intraocular pressure (IOP) or severe inflammatory reaction in traumatic cataract with posterior capsule rupture, delaying surgery would have allowed a more favorable intraocular lens (IOL) implantation in capsular bag with a better visual prognosis and lesser complications after control of inflammation and fibrosis of the capsule rupture [8].

To explore the possibility of minimal surgery in selected patients with small ferrous IVFD and traumatic cataract, here we report a series of cases with primary removal of IVFD by external magnetic extraction and secondary cataract removal combined with IOL implantation without PPV, which obtained good visual outcome without postoperative complications.

## Methods

The study comprised penetrating eyes with paracentral or peripheral self-sealing corneal penetrating wound, traumatic cataract and ferrous IVFD from September 2015 to March 2017. Eyes with endophthalmitis, retinal injury, vitreous hemorrhage, lens materials into the anterior chamber or vitreous cavity, active inflammation, or associated glaucoma were excluded from the study. The study was performed in accordance with the ethical standards stated in the Declaration of Helsinki.

A thorough history was collected from the patients. Best-corrected visual acuity (BCVA), slit-lamp examination, IOP, binocular indirect ophthalmoscopy wherever possible, B-scan ultrasonography, and orbital computed tomography were performed to evaluate the eye injuries.

After confirming that there was no secondary glaucoma, endophthalmitis, or retinal injury and identification of the metallic-like foreign body suspended in the vitreous cavity, primary removal of IVFD was performed by direct external magnetic extraction. The pars plana, adjacent to the foreign body, was exposed by opening the bulbar conjunctiva. Preplacing sclerotomy suture was performed to allow quick closure once the foreign body was removed. A sclerotomy, 4.0 mm from the corneal limbus, was made. The choroid was diathermized and incised. The rare earth magnet was placed at the sclerotomy site and the foreign body was removed. The vitreous, if attached to the foreign body, was cut before the foreign body left the sclera. The sclerotomy and the open conjunctiva were closed by 8–0 absorbable polypropylene suture (W9560, Johnson & Johnson). After removal of the IVFD, patient received 0.5% levofloxacin eye drops, 1% prednisolone acetate eye drops and 1% pranoprofen eye drops 2–8 times a day for 4 weeks, and 0.5 g levofloxacin tablet a day for 4 days. Patients were followed up at 1 day, 3 days, 1 week, 1 month after surgery. Slit-lamp examination, IOP, binocular indirect ophthalmoscopy wherever possible, and B-scan ultrasonography were performed to exclude secondary glaucoma, endophthalmitis or retinal detachment.

Two to 5 months after primary removal of the IVFD, the traumatic cataract was removed by phacoemulsification and/or aspiration (Bausch & Lomb, Stellaris). In white cataract, for a better view of the anterior capsule, 0.5% indocyanine green was applied for the staining of the anterior capsule. To prevent an enlargement of the posterior capsule rupture and vitreous prolapse, bottle height was set at 80 cm and maximal vacuum was set at 250 mmHg, viscoelastic (Healon, Johnson & Johnson Vision) was injected into the anterior chamber before withdrawal of the ultrasonic or irrigation/aspiration handle. If necessary, anterior vitreous vitrectomy was applied. IOLs (8 from AMO, AR40e and 4 from AMO, ZCB00) were placed in the capsule bag or in the ciliary sulcus. Postoperatively, patient received 0.5% levofloxacin eye drops 4 times a day for 2 weeks, 1% prednisolone acetate eye drops and 1% pranoprofen eye drops 2–4 times a day for 8 weeks. Patients were followed up at 1 day, 1 week, 1 month, 3 months, 6 months and 12 months after surgery.

## Results

**Fig. 1:**
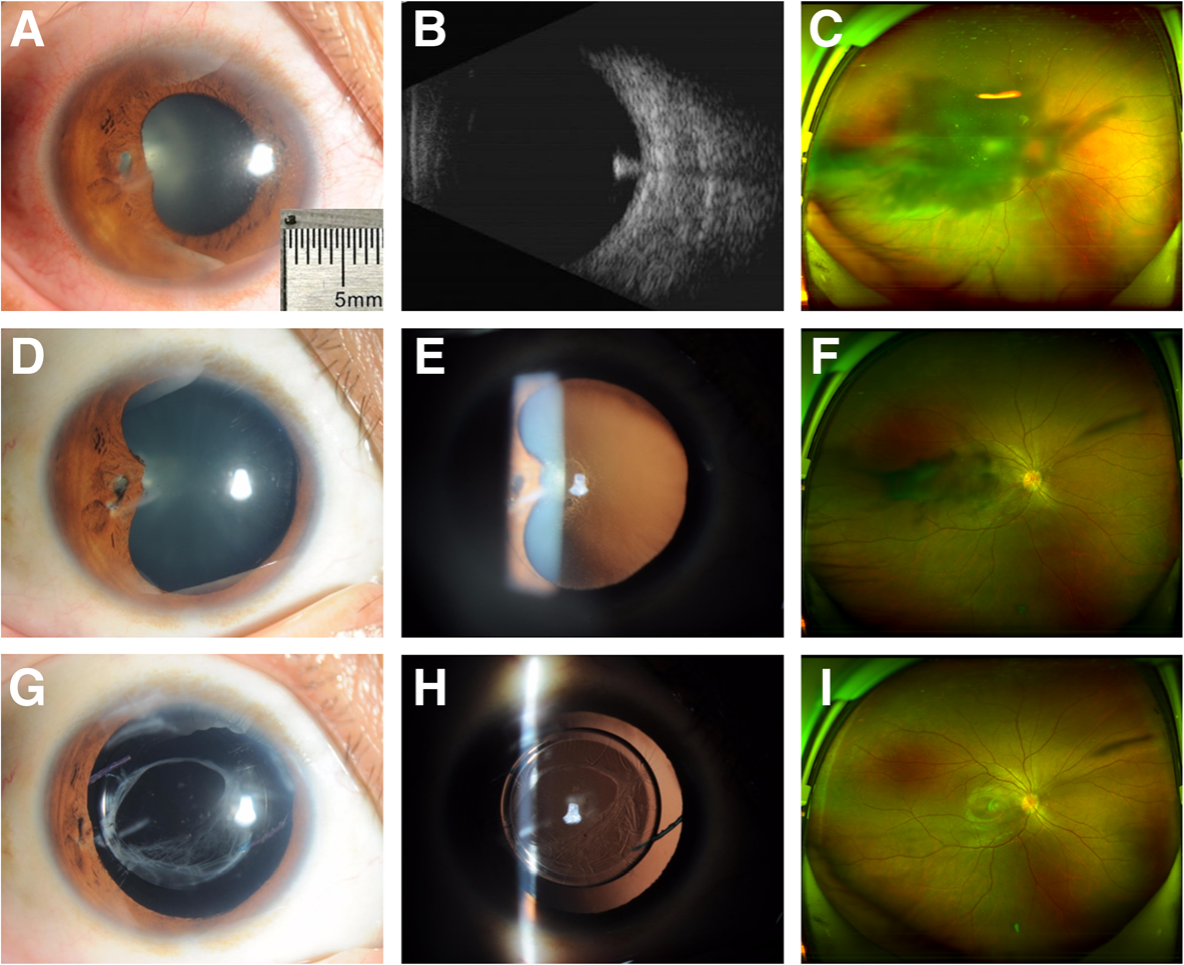
Patient 6 with localized cataract. **a**: Anterior segment photograph revealed a paracentral self-sealing corneal penetrating wound at 8 o’clock position, iris defect and posterior synechia, and localized cataract involved the visual axis. The size of foreign body was 1.0 mm in width and 1.0 mm in length. **b**: A small metallic-like foreign body was identified by B-scan ultrasonography. **c**: A dense shadow appeared in the middle of visual field by scanning laser ophthalmoscopic image. **d**, **e**: The traumatic cataract partially resolved 5 months after removal of the foreign body. **f**: A slight shadow appeared in the middle of visual field. **g**, **h**: The IOL was well centered 12 months after secondary removal of traumatic cataract and implantation of IOL. **i**: A linear shadow caused by the corneal scar appeared in peripheral visual field

**Fig. 2:**
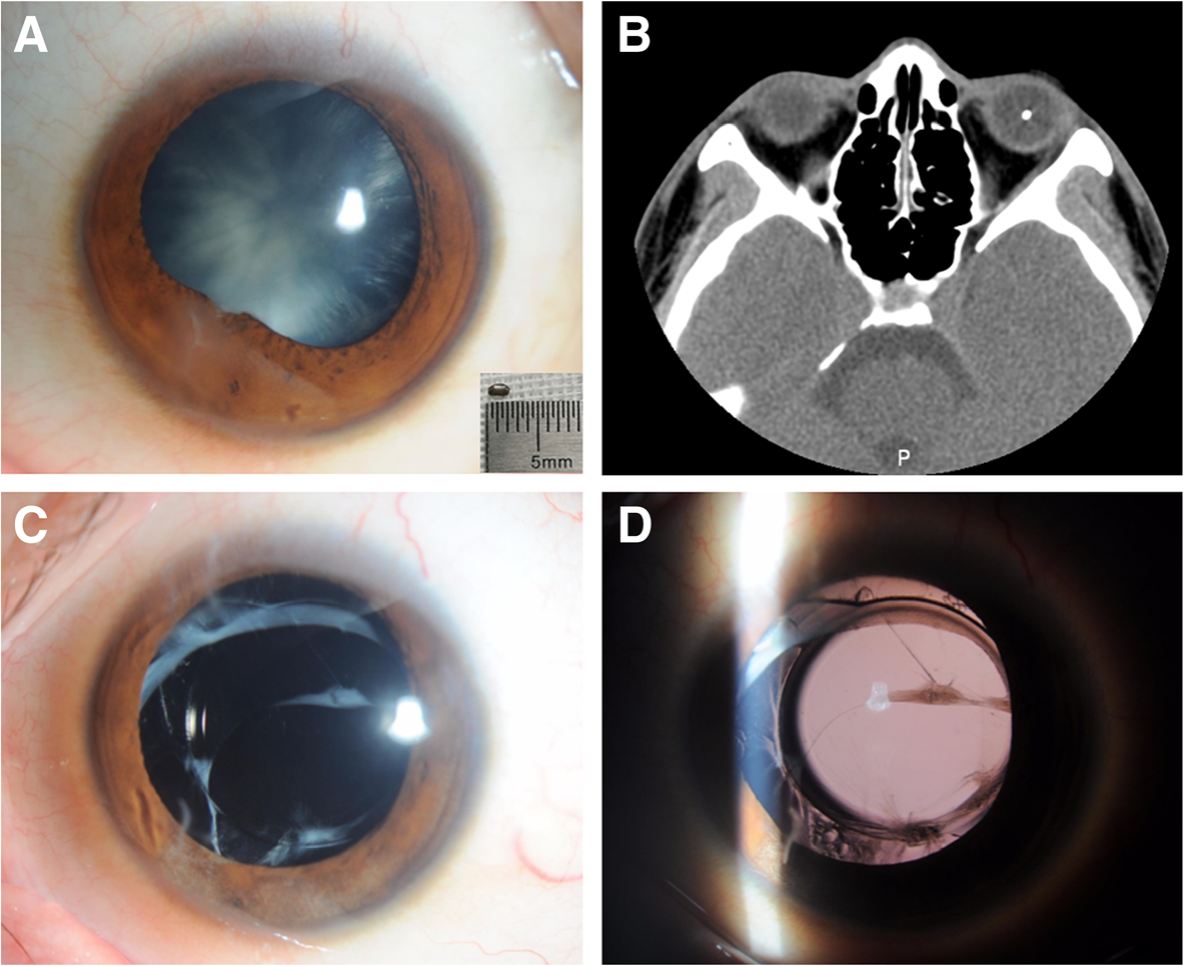
Patient 9 with total cataract. **a**: Anterior segment photograph revealed a peripheral self-sealing corneal penetrating wound at 7 o’clock position, iris posterior synechia, and total cataract. The size of foreign body was 1.5 mm in width and 2.0 mm in length. **b**: A small metallic-like foreign body, suspended in the vitreous cavity, was identified by orbital computed tomography. **c**, **d**: The IOL was well centered 12 months after secondary removal of traumatic cataract and implantation of IOL

Twelve patients (all men) were included in the study. The means of injury was hammering metal during occupational activities. The characteristics and outcomes are shown in Table 1. The average age was 32 years (range 19–46 years). The foreign bodies passed through cornea, iris, lens, and finally localized in the vitreous cavity. Four eyes developed localized cataract (Patient 6 with localized cataract was shown in Fig. 1), which interfered with visual axis, and 8 eyes developed total cataract (Patient 9 with total cataract was shown in Fig. 2).

**Table 1:** Demographics data and outcomes of all patients

NO	Age (ys)	Entry site	Cataract	Size of FD (mm)	Time to FD removal	Time to cataract surgery	IOL position	Follow up	BCVA	
									preo	final
1	34	C/I/L	localized	1.0 × 1.5	92 hs	142 ds	CB	15 ms	20/200	20/20
2	22	C/I/L	localized	1.0 × 2.0	68 hs	130 ds	CB	16 ms	20/200	20/25
3	30	C/I/L	total	1.5 × 3.0	38 hs	94 ds	CS	18 ms	HM	20/32
4	44	C/I/L	total	1.5 × 2.0	47 hs	98 ds	CB	15 ms	FC	20/25
5	46	C/I/L	localized	1.5 × 1.5	62 hs	125 ds	CB	18 ms	20/125	20/25
6	26	C/I/L	localized	1.0 × 1.0	70 hs	162 ds	CB	12 ms	20/100	20/20
7	24	C/I/L	total	1.5 × 2.0	20 hs	79 ds	CB	15 ms	FC	20/25
8	31	C/I/L	total	1.0 × 2.5	26 hs	90 ds	CB	12 ms	HM	20/32
9	19	C/I/L	total	1.5 × 2.0	40 hs	88 ds	CB	12 ms	FC	20/20
10	33	C/I/L	total	1.5 × 2.5	30 hs	79 ds	CS	12 ms	HM	20/32
11	42	C/I/L	total	1.0 × 2.0	44 hs	68 ds	CB	20 ms	FC	20/25
12	34	C/I/L	total	1.0 × 1.5	30 hs	75 ds	CB	10 ms	20/200	20/32

Abbreviations: *ys* years, *C* cornea, *I* iris, *L* lens, *FD* foreign body, *hs* hours, ds-days *IOL* intraocular lens, *CB* capsule bag, *CS* ciliary sulcus, ms-months, *BCVA* best corrected visual acuity, preo-preoperative, *HM* hand movement, *FC* finger counting

All cases presented self-sealing corneal penetrating wound, mild anterior chamber reaction, small iris defect and/or posterior synechia, and traumatic cataract without lens material into the anterior chamber or vitreous cavity. No secondary glaucoma was found. Metallic-like foreign bodies suspended in the vitreous cavity were identified by B-scan ultrasonography or orbital computed tomography. Small metallic-like IVFD was observed by indirect ophthalmoscopy in 2 patients with localized cataract. No retinal injury was found by B-scan ultrasonography.

BCVA ranged from hand movement to 20/100 before surgery. Time between injury and primary removal of foreign body was 47 h on average (range 20–92 h). All foreign bodies were successfully removed by external magnetic extraction through the pars plana incision. The mean size of the foreign bodies was 1.3 mm in width (range 1.0–1.5 mm) and 2.0 mm in length (range 1.5–3.0 mm). During follow-up, none of the secondary glaucoma, endophthalmitis or retinal detachment was found.

The average time between primary removal of foreign body and secondary traumatic cataract surgery was 107 days (range 79–162 days). For a better view of the anterior capsule, indocyanine green staining of the capsule was used in 8 patients with total cataract; anterior capsulorhexis was successfully performed in all cases. The cataract was removed by phacoemulsification and/or aspiration without significant enlargement of posterior capsule rupture in 10 patients, and the IOLs (6 from AMO, AR40e and 4 from AMO, ZCB00) were placed in the capsule bag. Enlargement of the posterior rupture was found in 2 patients (patient 3 and 10) with relatively large foreign bodies, anterior vitrectomy was performed and the IOLs (AMO, AR40e) were placed in the ciliary sulcus.

The mean time of follow-up was 15 months (range 12–20 months). BCVA ranged from hand movement to 20/100 before surgery and improved ranging from 20/32 to 20/20 at the final follow-up. The IOLs were well centered. Complications such as secondary glaucoma, endophthalmitis and retinal detachment were not found.

## Discussion

The prognosis of traumatic eye injuries associated with IOFB and traumatic cataract varies greatly depending on a number of factors, which include the time between trauma and IOFB extraction, initial visual acuity, entrance wound location, nature of IOFB, location of IOFB, preoperative retinal detachment, presence of intraocular hemorrhage, presence of endophthalmitis, primary surgical repair combined with IOFB removal and the occurrence of postoperative complications. Combined phacoemulsification, vitrectomy, foreign-body extraction, and IOL implantation have become more and more popular in the management of such patients [1, 10–12]. However, small ferrous IVFD can be successfully removed by external magnetic extraction through the pars plana incision in patients without endophthalmitis and retinal injury [7]. Secondary removal of traumatic cataract combined with IOL implantation without PPV has been reported [8]. In this study, we explore the possibility of minimal surgery in selected patients by primary removal of small ferrous IVFD by external magnetic extraction and secondary removal of traumatic cataract by phacoemulsification and IOL implantation without PPV, and obtain good visual outcomes without postoperative complications.

Advances in vitreoretinal instruments and surgical techniques have improved the success of treatment in eye injuries with posterior segment IOFBs. Removal of posterior segment IOFBs by PPV is the main surgical procedure that provides direct viewing and controlled surgery [8]. However, removal of the posterior hyaloid, an important surgical goal, is difficult in relatively young patients. Various potential complications of PPV have been reported, including iatrogenic retinal tears, suprachoroidal hemorrhage, hypotony, choroidal detachments, wound leaks, vitreous incarceration and drop of foreign body on the macula [4, 9, 13]. In selected patients with small ferrous foreign body positioned in the vitreous cavity and without endophthalmitis and retinal injury, removal of the IVFD by external magnetic extraction through the pars plana incision may obtain good outcomes without complications [7]. In this study, all of IVFDs were successfully removed by external magnetic extraction without complications.

Lenticular injury as a result of an IOFB may occur directly if the foreign body passes through the lens. Removal of the IOFB by PPV in the presence of traumatic cataract and associated retinal pathology is difficult. To allow clear visualization of the posterior segment, cataract extraction under such circumstance is often necessary. However, in a few cases, a minor injury to the lens may result in a localized nonprogressive lens opacity that does not require surgery. Small IOFB with limited capsular damage may lead to self-limited lens injury [14], and spontaneous resolution of a traumatic cataract after removal of an intralenticular foreign body has been reported [15]. In one of our patients, traumatic lens opacity was mostly resolved and did not interfere with the visual axis after removal of a ferrous IVFD by external magnetic extraction, and the lens was preserved (unpublished data).

In the absence of lens material into the anterior chamber in traumatic cataract, which may cause increased IOP or severe inflammatory reaction, some studies state that it is better to treat the eye with topical steroids to control inflammation first and to allow the capsule fibrosis. Delaying surgery would have allowed a more favorable implantation of the IOL in capsular bag with a better visual prognosis and lesser complications.

In this study, after primary removal of the IVFD, patients received systemic and topical antibiotic to prevent infection and topical steroids to control inflammation. Secondary cataract removal by phacoemulsification and IOL implantation in the capsule bag were successfully performed in 10 patients without significant enlargement of posterior capsule rupture. In 2 patients with relatively large foreign bodies, enlargement of the posterior rupture was inevitable, thus, anterior vitrectomy was performed and IOLs were placed in the ciliary sulcus. During follow-up, BCVA significantly improved, ranging from 20/32 to 20/20, and the IOLs were well centered. Complications, such as secondary glaucoma, endophthalmitis and retinal detachment were not observed.

## Conclusions

In conclusion, in selected penetrating eyes with small ferrous IVFD combined with traumatic cataract without retinal injury, secondary glaucoma and endophthalmitis, primary removal of the IVFD by external magnetic extraction followed by secondary cataract removal and IOL implantation is an appropriate choice. Minimal surgery may obtain good visual outcomes without complications.

## Abbreviations

BCVA: Best-corrected visual acuity
IOFB: Intraocular foreign body
IOL: Intraocular lens
IOP: Intraocular pressure
IVFD: Intravitreal foreign body
PPV: Pars plana vitrectomy
